# Strigolactones initiate the formation of haustorium-like structures in *Castilleja*

**DOI:** 10.1016/j.isci.2024.111491

**Published:** 2024-11-28

**Authors:** Marco Bürger, Danica Peterson, Joanne Chory

**Affiliations:** 1 Plant Biology Laboratory, Salk Institute for Biological Studies, 10010 North Torrey Pines Road, La Jolla, CA 92037, USA; 2 Howard Hughes Medical Institute, Salk Institute for Biological Studies, 10010 North Torrey Pines Road, La Jolla, CA 92037, USA

**Keywords:** Plant biology, Plant Genetics, Plant physiology

## Abstract

Strigolactones serve as germination signals for several root-parasitic plant species within the Orobanchaceae family. Yet, their role in the life cycle of the facultatively parasitic genus *Castilleja* has remained elusive. Here, we demonstrate that strigolactones initiate the formation of haustorium-like structures in *Castilleja*. We present the genome of *Castilleja foliolosa*, which reveals an abundance of KAI2d paralogs that act as strigolactone receptors. *Castilleja foliolosa*’s KAI2d proteins include high-turnover enzymes, in particular KAI2d15, which features a substrate binding pocket with a methionine cluster at its entrance that is involved in the trade-off between substrate turnover and affinity. Our findings provide insights into strigolactone perception in *Castilleja* and refine our perspective on their role in facultative parasitism.

## Highlights

• Strigolactones alter normal root development in *Castilleja* seedlings
• *C. foliolosa* genome contains 15 kai2d paralogs as potential strigolactone receptors
• Tested strigolactone receptors show high catalytic efficiency
• Results provide insights into strigolactone perception in facultative parasite *Castilleja*

## Graphical abstract

**Figure:**
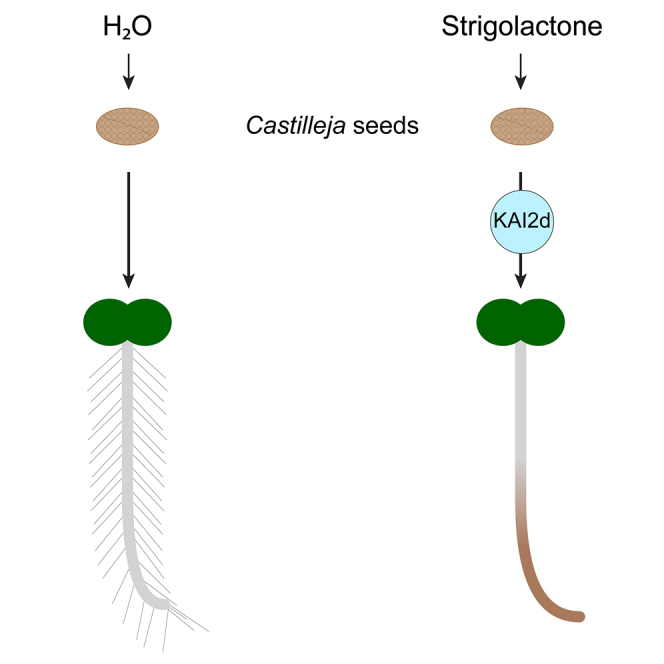

## Introduction

The Orobanchaceae family encompasses a diverse array of mostly parasitic plant species, which, after major rearrangements in the Lamiales, includes the genus *Castilleja*, commonly known as Indian Paintbrush.^1^^,^^2^ With a distribution spanning the Americas and some species documented in Asia and Russia, *Castilleja* comprises over two hundred annual and perennial herbaceous species.^3^ Host dependence within the Orobanchaceae family exhibits a wide spectrum. *Striga* species, for example, are obligate hemiparasites that rely on hosts for survival while retaining photosynthetic capabilities. In contrast, *Orobanche* species exemplify holoparasitism, entirely dependent on hosts for sustenance due to the loss of photosynthetic function.^4^ *Phtheirospermum japonicum*, *Triphysaria,* and *Castilleja*, on the other hand, are facultative parasites, capable of transitioning between autotrophic and hemiparasitic lifestyles based on environmental conditions. Root-parasitic plants have developed a specialized organ, the haustorium, to establish connections with the host’s vascular system.^5^^,^^6^ The haustorium allows for the transfer of water and nutrients from the host to the parasitic plant, enabling their exploitation of host resources and successful adaptation to nutrient-deficient environments. Within the life cycle of Orobanchaceae, the detection and utilization of host-derived signaling molecules play a key role in the parasitic lifestyle: Notably, strigolactones, a group of carotenoid-derived plant hormones, are well-known for their role in inducing germination of seeds from obligatory parasitic Orobanchaceae species.^7^^,^^8^^,^^9^ Many parasitic Orobanchaceae have evolved a large clade of diversified KAI2 proteins (KAI2d) as receptors for exogenous strigolactones. The diversification of these proteins is regarded as the main driver of parasitism for these plants.^10^ While the host specificity of *Castilleja* species and the structure of the *Castilleja* haustorium have been extensively described,^11^^,^^12^^,^^13^^,^^14^^,^^15^^,^^16^ it is unclear if these plants can perceive strigolactones and whether strigolactones have any biological role in the parasitic lifestyle of *Castilleja*. As a matter of fact, it has been demonstrated that *Castilleja* seeds are not impacted by the hemiparasitic nature of *Castilleja*, but that germination occurs after a several weeks or months long moist chilling period.^17^

Here we show that strigolactones are perceived by multiple proteins of the diverged clade of KAI2 proteins and trigger the formation of haustorium-like structures in *Castilleja*.

## Results

### Strigolactones trigger the formation of haustorium-like root structures in *Castilleja*

**Figure 1:**
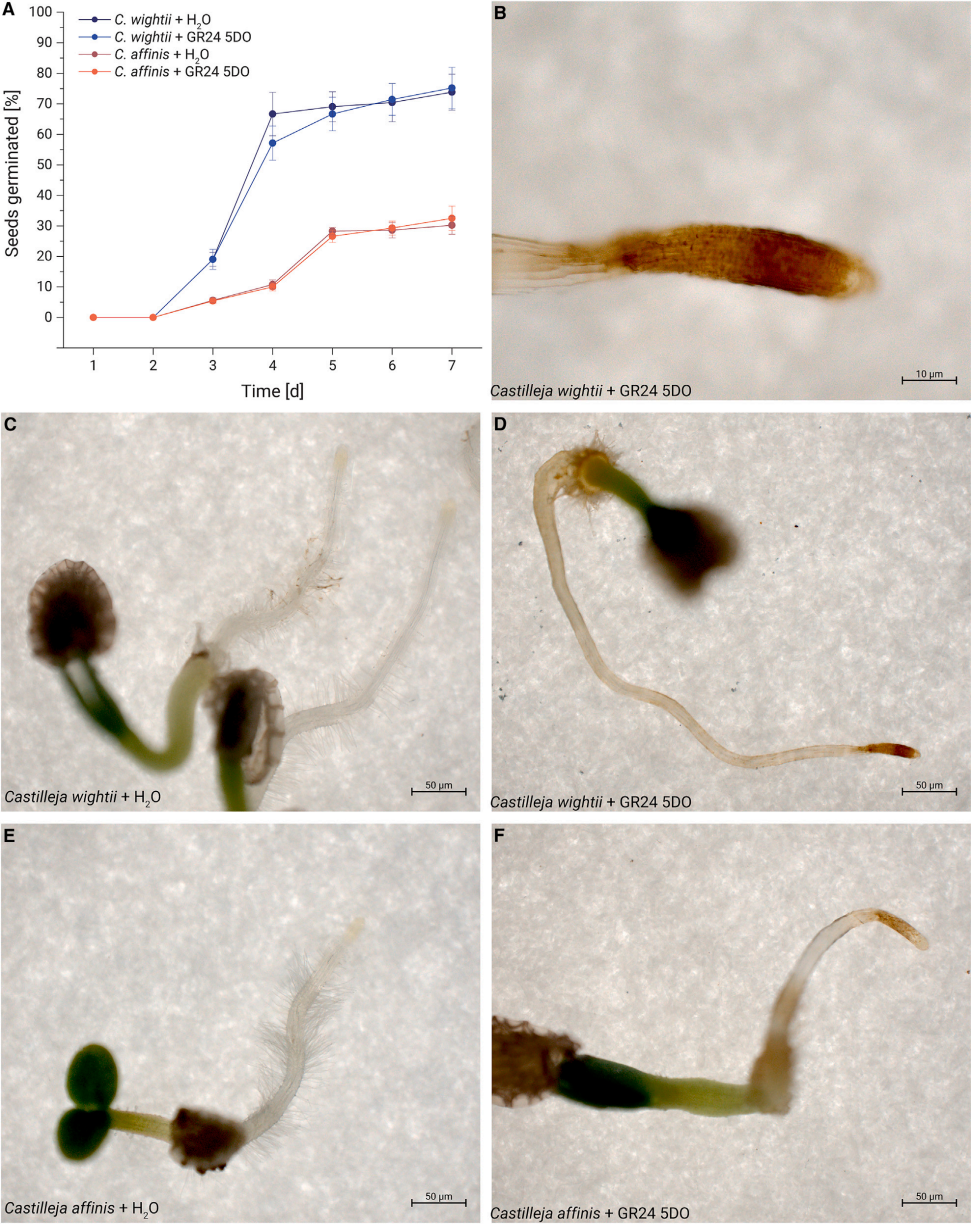
GR24 triggers the formation of haustorium-like structures in *Castilleja* (A) Germination assay showing percentages of germinated *C. wightii* and *C. affinis* seeds when germinated on H_2_O vs. GR24 5DO. Error bars represent the standard deviation of three independent measurements. (C and E) Heterotrophic root phenotypes and (B, D, and F) possibly parasitic phenotypes of *C. wightii* and *C. affinis* after germination on GR24 5DO.

We investigated the potential biological role of strigolactones in the life cycle of *Castilleja* species. To this end, we germinated stratified seeds from *Castilleja wightii*, *Castilleja affinis*, and *Castilleja foliolosa* under two different conditions: in the presence of H_2_O and in the presence of the chemical strigolactone analog GR24 5DO. Despite the different conditions, germination efficiency did not vary significantly between the two environments (Figure 1A). However, we noted marked morphological differences in the emergent seedlings. Seedlings that germinated in H_2_O developed a robust primary root system accompanied by an array of lateral root hairs (Figures 1C–1E and S1A). In contrast, the seedlings exposed to GR24 5DO presented a slenderer root structure devoid of lateral root hairs (Figures 1B, 1D, 1F, and S1B). This divergence in morphology was consistent across the three tested *Castilleja* species. We quantified the percentage of germinated seedlings that developed such root structure after exposure to different strigolactones but found no significant difference (Figure S2). When we examined the terminal root structure more closely, we noticed that GR24 5DO grown plants had a smaller root cap and smaller cells at the tip of the structure compared to water grown plants (Figures S1C–S1F). We used Rhodamine 123, which serves as an indicator for mitochondrial transmembrane potential, to confirm the viability of these cells (Figures S1C–S1F). In summary, the structures grown in the presence of GR24 5DO more closely resemble the elongated state characteristic of root-parasitic plants such as *Striga* than they do typical roots.

These observations suggest that while strigolactones may not function as a germination signal for *Castilleja* species, they do effectively mimic a host plant, participating in triggering a parasitic state in the seedlings immediately post-germination when present. Therefore, strigolactones appear to play a crucial role in activating post-germination modifications in *Castilleja*, preparing for a parasitic lifestyle. Considering the impact of strigolactones on the morphological development of *Castilleja*, we set out to identify the strigolactone receptors within *Castilleja foliolosa* and aimed to characterize these proteins.

### Genome sequence of *Castilleja foliolosa* reveals 15 *kai2d* genes

To investigate the potential presence of strigolactone receptors in *Castilleja*, we sequenced the genome of *Castilleja foliolosa*, also known as Woolly Indian Paintbrush. The assembly spanned 551 contigs, adding up to a total length of approximately 752 Mbp with an N50 of 11,150,047 bp and an N90 of 2,884,952 bp (Table S1). The haploid genome length, as determined through a K-mer distribution analysis,^18^ measured approximately 555 Mbp, and a Smudgeplot analysis^18^ indicated that the genome is likely diploid (Figures S3A and S3B), which is in agreement with a previous study.^19^ BUSCO analysis against the eudicots_odb10 lineage dataset^20^ showed that 87.5% of the BUSCOs were complete (Table S2).

**Figure 2:**
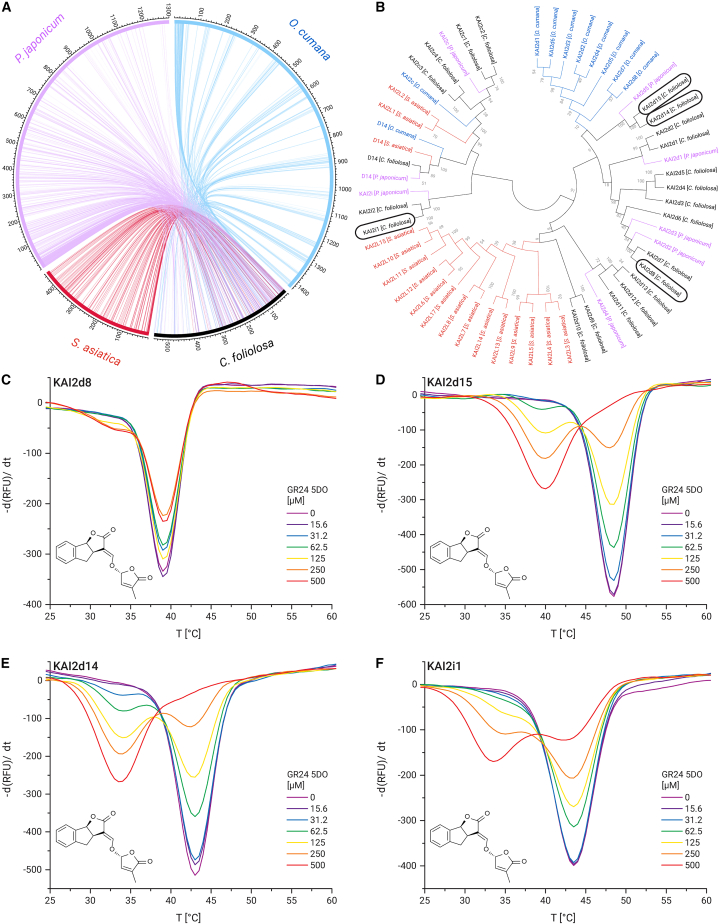
Strigolactone receptors in the genome of *Castilleja foliolosa* (A) Circos plot showing synteny between *C. foliolosa* and 3 other parasitic plants: The facultative hemiparasite *P. japonicum*, the obligate hemiparasite *S. asiatica*, and the holoparasite *O. cumana*. The scale has been adjusted for genome size and is in Mbp. The number of syntenies to *C. foliolosa* are 21,204 with *P. japonicum*, 15,251 with *O. cumana*, and 12,476 with *S. asiatica*. (B) Phylogenetic tree of D14, KAI2c, and KAI2d proteins from *C. foliolosa*, *P. japonicum*, *S. asiatica*, and *O. cumana*. Proteins in black boxes were used for differential scanning fluorimetry (DSF) as shown as follows: (C–F) DSF of *C. foliolosa* KAI2d8, KAI2d14, KAI2d15, and KAI2i1, showing their destabilization upon presence of the chemical strigolactone analog GR24 5DO. DSF curves are shown as mean values of three independent measurements.

We compared the genome of *Castilleja foliolosa* to the published genomes of other species within the Orobanchaceae family, namely *Phtheirospermum japonicum*,^21^ *Striga asiatica*,^22^ and *Orobanche cumana*.^23^ As anticipated, a substantial number of syntenies were found between *Castilleja foliolosa* and these other parasitic plant genomes, with the highest count of 21,204 syntenies noted with *Phtheirospermum japonicum*, followed by 15,251 syntenies with *Orobanche cumana* and 12,476 syntenies with *Striga asiatica* (Figure 2A).

Utilizing a BLAST search, potential strigolactone receptors were identified. We discovered 1 gene encoding for DWARF14, 4 genes encoding KAI2c paralogs, 2 genes encoding KAI2i proteins, and 15 genes encoding KAI2d proteins (Figure 2B). This high number of paralogs in the KAI2d protein clade is in line with other parasitic plants in the Orobanchaceae, suggesting a potential function as strigolactone receptors in *Castilleja* as well. We collected bulk RNA from *Castilleja affinis* and *Castilleja foliolosa* seedlings and conducted PacBio Iso-Seq to identify full-length transcripts. Additionally, we analyzed a publicly available RNA-seq dataset from *Castilleja miniata*. In all three species, we were able to detect the presence of transcripts from several kai2d orthologs, including those that we analyzed from *Castilleja foliolosa* (Figure S3C).

### KAI2d proteins from *Castilleja foliolosa* are putative strigolactone receptors

To further investigate if these predicted KAI2 proteins serve as strigolactone receptors, we heterologously produced five of them, namely KAI2d8, KAI2d9, KAI2d14, KAI2d15, and KAI2i1, in *Escherichia coli*. We were, unfortunately, unable to produce sufficient amounts of the other orthologs from *E. coli*. By conducting differential scanning fluorimetry (DSF), we observed a clear, concentration-dependent destabilization of KAI2d14, KAI2d15, and KAI2i1 in the presence of the chemical strigolactone analog GR24 5DO. A weaker but discernible destabilization was noted with KAI2d8, strongly suggesting that these KAI2 proteins are indeed strigolactone receptors (Figures 2C–2F). We were, unfortunately, unable to obtain sufficient amounts of KAI2d9 for the DSF assay.

### KAI2d15 features a methionine cluster at the substrate pocket entrance

**Figure 3:**
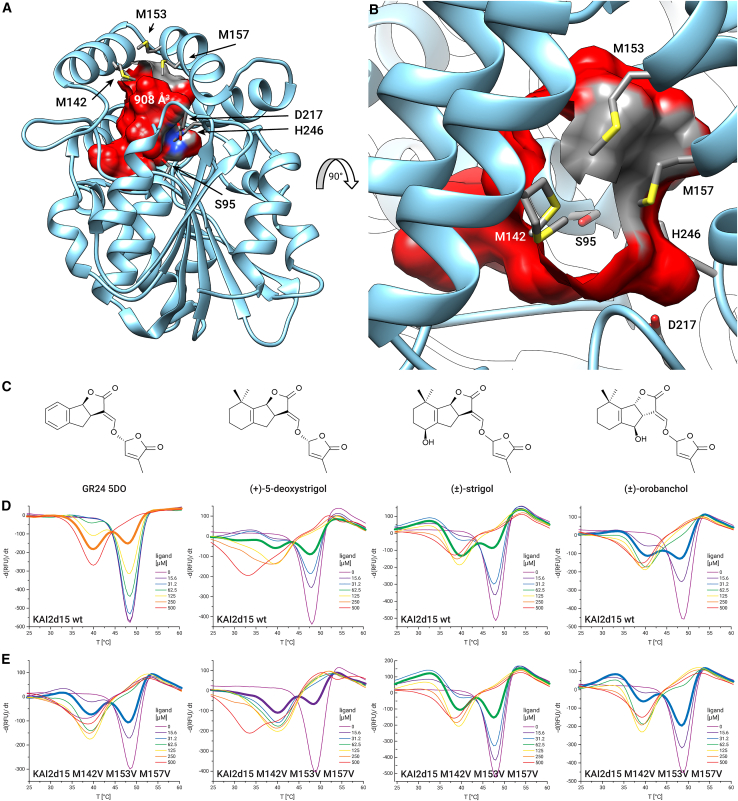
Structural and biochemical analysis of *C. foliolosa* KAI2d15 (A) Crystal structure of *C. foliolosa* KAI2d15, showing the substrate binding pocket in red, and highlighting the residues of the catalytic triad (S95, D217, H246), and the methionines surrounding the entrance to the pocket (M142, M153, M157). (B) Rotated and zoomed in view of the methionines surrounding the pocket entrance. Note that an alternative side-chain conformation of M142 is shown. (C) Chemical structures of the strigolactone molecules used in the following DSF assay. (D and E) DSF assay comparing the destabilization of *C. foliolosa* KAI2d15 wild type and a M142V M153V M157V variant upon the presence of different strigolactone molecules. Concentrations closest to the equilibrium are highlighted in bold for easier comparison. DSF curves are shown as mean values of three independent measurements.

To obtain additional insight into the strigolactone perception in *Castilleja*, we solved the crystal structure of KAI2d15 at a resolution of 1.8 Å (Table S3). As expected, the protein adopted a canonical α/β hydrolase architecture, with a 4-helix lid domain covering the substrate binding pocket containing a Ser/His/Asp catalytic triad at its base. The substrate binding pocket was quite voluminous, measuring 908 Å^3^ (Figure 3A), which surpassed the 861 Å^3^ found in the strigolactone receptor KAI2d4 from *Orobanche minor*,^24^ but was smaller than the 1111 Å^3^ detected in the highly sensitive SL receptor HTL7 from *Striga hermonthica*.^25^

We identified three methionine residues at the entrance to the binding pocket (Figure 3B). To study the effect of these methionine residues on the receptor’s substrate specificity and sensitivity, we created a protein variant, replacing the three methionines with valines (KAI2d15 M142V M153V M157V). We carried out separate DSF experiments with four different strigolactone molecules, testing both the variant and the wild-type protein. The mutant protein showed destabilization at lower ligand concentrations when GR24 or 5-deoxystrigol were present, while the wild-type protein destabilized at lower concentrations of strigol or orobanchol (Figures 3C–3E).

### Tested *Castilleja foliolosa* KAI2 proteins are high-turnover enzymes

**Figure 4:**
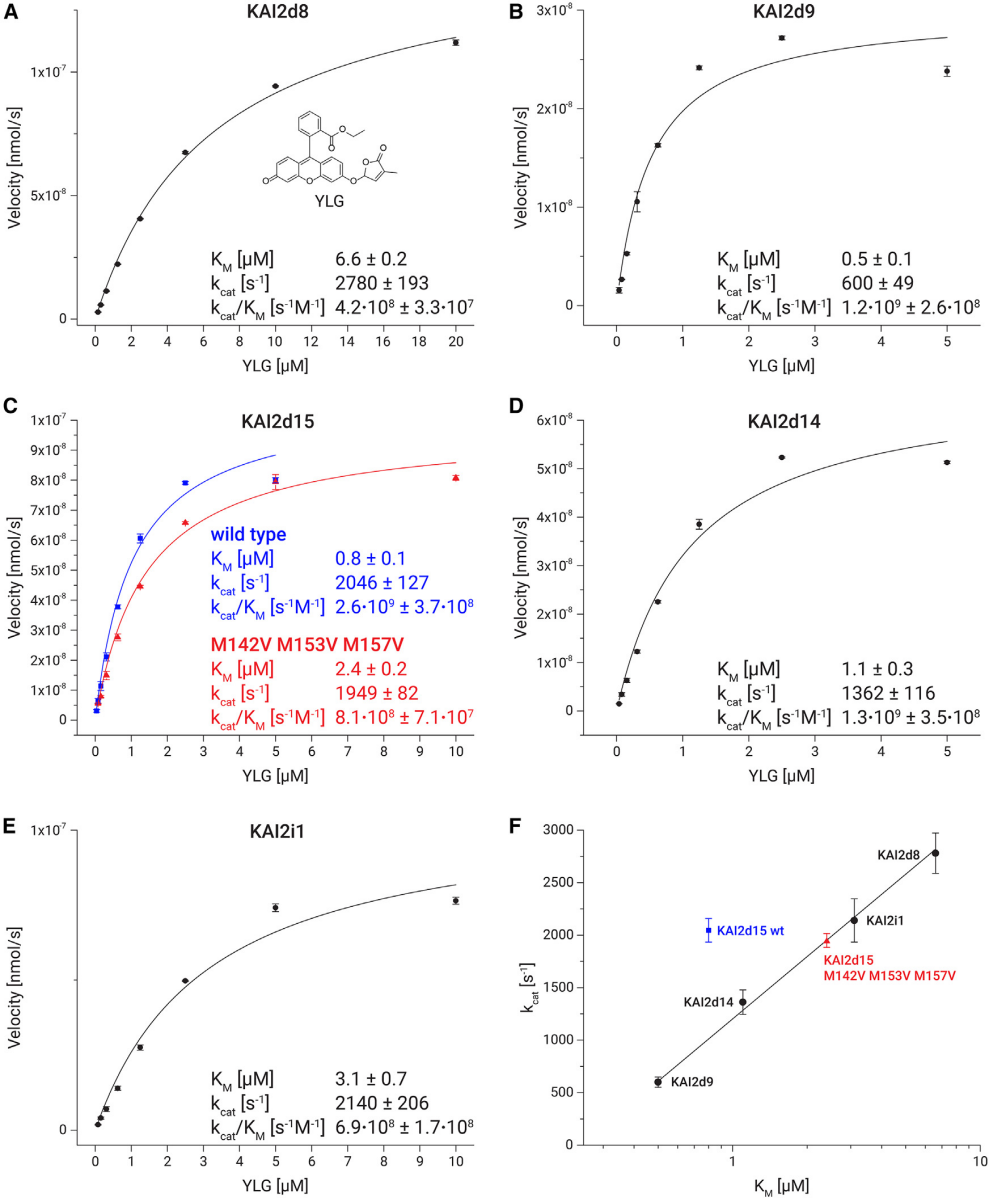
Michaelis-Menten assays of *C. foliolosa* KAI2d proteins with the fluorescent strigolactone analog Yoshimulactone Green (YLG) as substrate (A–E) Comparison of Michaelis-Menten values between different *C. foliolosa* KAI2 proteins (The chemical structure of YLG is shown in A). (F) Correlation between turnover rates and Michaelis constants of different *C. foliolosa* KAI2 proteins. Error bars represent the standard deviation of three independent measurements.

We examined the enzymatic properties of *Castilleja foliolosa* of KAI2d8, KAI2d9, KAI2d14, KAI2d15, and KAI2i1 in Michaelis-Menten experiments using the large fluorescent strigolactone analog Yoshimulactone Green (YLG). All investigated proteins turned out to be efficient high-turnover enzymes, with turnover numbers ranging between 600 s^−1^ and 2780 s^−1^ and catalytic efficiencies between 4.2·10^8^ s^−1^M^−1^ and 2.6·10^9^ s^−1^M^−1^ (Figures 4A–4E). We specifically compared Michaelis-Menten parameters between the KAI2d15 wild type and the mutant, in which three of the methionines at the substrate pocket entrance had been replaced with valines (KAI2d15 M142V M153V M157V). While the turnover numbers (k_cat_) of these proteins showed no significant difference, we found a 3-fold decrease in the Michaelis constant (K_M_) for wild-type KAI2d15 as compared to the mutant version (0.8 μM in wild-type KAI2 vs. 2.4 μM in KAI2d15 M142V M153V M157V) (Figure 4C). We further noticed a proportional relationship between the Michaelis constants and the turnover rates of these proteins (Figure 4F), suggesting a trade-off between substrate turnover and affinity in the catalytic reaction. KAI2d15 wild type was a notable exception, maintaining both high k_cat_ and low K_M_. Strikingly, the KAI2d15 M142V M153V M157V version of the protein lay well within the trendline shared with the other KAI2d proteins, as a result of the higher K_M_ value compared to wild type. These results suggest that the properties of the methionine residues located at the entrance to the substrate binding pocket allow KAI2d15 to overcome this trade-off between substrate turnover and affinity that appears to be characteristic of the other investigated KAI2d proteins from *Castilleja foliolosa*.

## Discussion

Strigolactones have long been recognized as germination signals for root-parasitic plants from the Orobanchaceae family. Beyond this, they can also function as chemoattractants for host tropism.^26^ While strigolactones serve as potent germination stimulants for obligate parasites such as *Striga* and *Orobanche*, their role is different in the facultative parasite *Phtheirospermum japonicum*, where they stimulate germination only in the absence of nitrate ions.^27^ In our study, we discovered a role for strigolactones in *Castilleja*, another facultative parasite. Here, strigolactones do not cause germination but instead act post-germination. *Castilleja* can adopt a parasitic lifestyle when their root system encounters a host organism – a behavior well documented in adult plants, which form lateral haustoria upon exposure to a host.^13^ However, there is no data supporting that strigolactones are involved in the parasitic transition. We demonstrate that the presence of strigolactones during germination suppresses the development of a typical root system and instead leads to the formation of a structure reminiscent of the elongated stage known from other root-parasitic plants, possibly representative of a parasitic state in *Castilleja*. This adds a layer of complexity to our understanding of the functions of strigolactones within the Orobanchaceae family.

The results of our syntenic block analysis and sequence comparison provide insights into the genomic relationships among the four species of plants studied, which include *Orobanche cumana*, *Castilleja foliolosa*, *Striga asiatica*, and *Phtheirospermum japonicum*. All these species belong to the Orobanchaceae family and display parasitic lifestyle traits; however, the degree and nature of parasitism vary among them. *Castilleja foliolosa* and *Phtheirospermum japonicum*, both facultative hemiparasites, shared the highest number of syntenic blocks, indicating a closer genomic relationship, contrasting with *Orobanche cumana* and *Striga asiatica*, which have different parasitic strategies. When we examined the strigolactone receptor sequences, a family of genes known for their rapid evolution and critical role in plant parasitism, we found that the receptors in *Castilleja foliolosa* are most closely related to those in *Phtheirospermum japonicum*. This provides additional evidence of a closer evolutionary relationship between these two species, and it further suggests that their shared facultative parasitic lifestyle has them placed closer to their common ancestor than obligatory parasites in the Orobanchaceae, which are assumed to have evolved later.^4^

We identified 15 paralogs of the diverged clade KAI2 proteins (KAI2d) encoded in the genome of *Castilleja foliolosa*, one of which, KAI2d15, showed characteristics previously not observed in a strigolactone receptor. We found 3 methionine residues in the crystal structure of KAI2d15 that surround the entrance to the protein’s substrate binding pocket, including an alternative side-chain conformation of M142. We speculate that there might be a role of the branched, flexible methionines in the more favorable accommodation of strigol and orobanchol, which are slightly larger than GR24 and 5-deoxystrigol. The methionines also appear to optimize substrate affinity and turnover. In many enzymes, achieving a high affinity for a substrate often comes at the cost of efficient substrate turnover. This is largely due to the binding dynamics: a tightly bound substrate might be converted efficiently but released slowly, leading to a longer turnover cycle.^28^ We observed a linear trade-off between these two catalytic parameters in all investigated KAI2 proteins, except KAI2d15, and remarkably, the trade-off was restored in the KAI2d15 M142V M153V M157V mutant. Flexible methionine residues at the pocket entrance might aid substrate translocation, resulting in a lower K_M_. However, their flexibility could also play a role in destabilizing the enzyme-product interaction post-catalysis, making product release more efficient and maintaining a higher k_cat_. Thus, a possible dual role of the methionines in both enhancing substrate binding and promoting product release might be the molecular basis for KAI2d15 mitigating the typical trade-off observed in enzyme kinetics. The pocket entrance methionine residues might also affect the affinity for GR24 specifically. The sulfur atom in methionine is capable of engaging in various types of interactions with aromatic moieties, including van der Waals forces and sulfur-π interactions. However, a comprehensive understanding of these interactions would necessitate studying a broader variety of strigolactones to discern the specificity and affinity nuances. The presence of methionines in the entrance to the SL binding pocket has been previously reported in the case of *Striga hermonthica* HTL4.^25^ While two of the methionines in the pocket entrance in *Castilleja foliolosa* KAI2d15 align with methionines in ShHTL4, the third methionine (M157) corresponds to a phenylalanine in ShKAI2d4 (F157) (Figure S4A). It is also noteworthy that none of the *Striga hermonthica* or *Striga asiatica* KAI2d proteins contain methionine at this position. A different methionine in ShKAI2d4 (M154) is located deeper inside the pocket, rather than at its entrance. In *Striga hermonthica*, the presence or absence of a crucial hydrogen bond between F150/Y151 and L178 determines substrate specificity.^25^ This structural basis, however, does not seem to be applicable to the protein we investigated. *Castilleja foliolosa* KAI2d15 features a histidine instead of a phenylalanine/tyrosine at this position. In addition, this histidine is about 4.7 Å away from the corresponding leucine, likely too distant for hydrogen bonding (Figure S4B).

A notable observation is the generally high turnover rates exhibited by *Castilleja foliolosa*’s KAI2 proteins. While the turnover rates of SL receptors vary, especially D14 proteins are characterized by slow or even single-turnover rates.^29^^,^^30^^,^^31^ The proteins in this study demonstrated substantial catalytic efficiencies, the highest achieved by KAI2d15 at 2.6·10^9^ s^−1^M^−1^ on YLG, which is in the range of a diffusion-limited enzyme. While it is not immediately clear whether there are advantages to high-turnover SL receptors, it seems intuitive that such a receptor, which rapidly hydrolyzes strigolactones, would depend on constant strigolactone input to maintain its activation and availability for binding to the F box protein MAX2, the next step in the strigolactone signaling pathway after SL perception by its receptor.^8^ Diverse strigolactone turnover rates through different receptors could possibly enable the plant to map out strigolactone gradients in its surroundings. Another reason for the elevated catalytic activity observed in certain KAI2d proteins could be a decrease in strigolactone sensitivity. This could be through either the degradation of the host’s ligand before signal transduction begins or by necessitating elevated levels of strigolactone for a prolonged signaling event to take place. Additionally, it might be beneficial for a hemiparasite to either eliminate or disregard its own strigolactone production in the root area.

In summary, our research reveals that *Castilleja* possesses a large clade of KAI2d proteins, acting as strigolactone receptors, participating in activating a parasitic state in the plant. Notably, one of these receptors operates as a diffusion limited enzyme that achieves specificity for larger strigolactones and high catalytic efficiency through a cluster of methionine residues at the pocket entrance. Hopefully, in the future, transgenic *Castilleja* plants will be available to study the function of these receptors in isolation and *in planta*. We hope that our findings will lay the groundwork for future studies investigating the role of strigolactones in *Castilleja*.

### Limitations of the study

Our study reveals important insights into strigolactone perception in *Castilleja* and opens avenues for future research. The development of a reliable transformation system for *Castilleja* species would allow the validation of our findings through genetic approaches, such as knockout studies of individual kai2d genes. Among the fifteen identified KAI2d paralogs, we successfully characterized five through biochemical analyses, leaving opportunities to explore the properties of additional family members as expression systems are optimized. While we observed clear morphological changes in seedling root structures upon strigolactone treatment, future studies can further illuminate the cellular and molecular nature of these structures and their relationship to parasitic states. These considerations highlight promising directions for future studies that will further expand our understanding of strigolactone signaling in facultatively root-parasitic plants.

## Resource availability

### Lead contact

Further information and requests for resources should be directed to and will be fulfilled by the lead contact, Marco Bürger (mburger@salk.edu).

### Materials availability

This study is based on existing materials and did not produce new materials or reagents.

### Data and code availability

• The genome sequence of *Castilleja foliolosa* has been deposited in the NCBI GenBank under accession number GenBank: JAVIJP000000000.
• The structural coordinates and diffraction data of *Castilleja foliolosa* KAI2d15 have been deposited in the Protein DataBank under accession code PDB: 8TMX.
• Data reported in this article will be shared by the lead contact upon request.
• This article does not report the original code.
• Any additional information required to reanalyze the data reported in this work article is available from the lead contact upon request.

## Acknowledgments

We extend our gratitude to Dr. Xuelin Wu (Salk Institute for Biological Studies) for the generous help with microscopy. This work was supported by the NGS Core Facility of the Salk Institute with funding from NIH-NCI CCSG: P30 014195, the Chapman Foundation and the Helmsley Charitable Trust. We thank the staff at Advanced Light Source at the Berkeley Center for Structural Biology for their assistance with X-ray data collection. The Berkeley Center for Structural Biology was supported in part by the NIH, National Institute of General Medical Sciences and the Howard Hughes Medical Institute. The Advanced Light Source was supported by the Director, Office of Science, Office of Basic Energy Sciences of the U.S. Department of Energy (contract no. DE-AC0205CH11231). This study has been supported by the 10.13039/100000002National Institutes of Health (NIH) grant R35 GM122604. Joanne Chory is an investigator of the Howard Hughes Medical Institute.

## Author contributions

M.B. and D.P. conducted the experiments. All authors wrote the article.

## Declaration of interests

The authors declare no conflicts of interest.

## STAR★Methods

### Key resources table

**Table:** 

REAGENT or RESOURCE	SOURCE	IDENTIFIER
**Bacterial and virus strains**		

BL21-CodonPlus (DE3)-RIL competent cells	Agilent	230245

**Chemicals, peptides, and recombinant proteins**		

GR24 5DS	Strigolab	EN1010
Rhodamin 123	Sigma-Aldrich	6030951
Isopropyl β-D-1-Thiogalactopyranosid, IPTG, Isopropyl β-D-Thiogalactosid (IPTG)	Sigma-Aldrich	I6758
Tris(2-carboxyethyl)phosphin -hydrochlorid (TCEP)	Sigma-Aldrich	C4706
HRV 3C Protease	Takara	7360
SYPRO Orange	ThermoFisher	S6651

**Critical commercial assays**		

Nanobind plant nuclei kit	PacBio	102-207-800
SMRTbell Express Template Prep Kit 2.0	PacBio	100-938-900

**Deposited data**		

Genome sequence of Castilleja foliolosa	GenBank	JAVIJP000000000
Crystal structure of *Castilleja foliolosa* KAI2d15	Protein Data Bank	8TMX

**Recombinant DNA**		

pGEX-4T-1 Castilleja foliolosa kai2d8	Genscript (this study)	
pGEX-4T-1 Castilleja foliolosa kai2d9	Genscript (this study)	
pGEX-4T-1 Castilleja foliolosa kai2d14	Genscript (this study)	
pGEX-4T-1 Castilleja foliolosa kai2d15	Genscript (this study)	
pGEX-4T-1 Castilleja foliolosa kai2d15 M142V M153V M157V	Genscript (this study)	
pGEX-4T-1 Castilleja foliolosa kai2i1	Genscript (this study)	

**Software and algorithms**		

ZEN 3.7	Zeiss	
Hifiasm 0.16.0	https://github.com/chhylp123/hifiasm	Cheng et al.^27^
Helixer v0.3.3	https://github.com/weberlab-hhu/helixer	Stiehler et al.^28^
InterProScan 5.55-88.0	https://github.com/ebi-pf-team/interproscan	Jones et al.^29^
EggNOG-mapper 2.1.12	https://github.com/eggnogdb/eggnog-mapper	Cantalapiedra et al.^30^
SynMap2	https://genomevolution.org/SynMap.pl	Haug-Baltzell et al.^31^
R 4.2.0	https://www.r-project.org/	R-Core-Team^32^
MEGA11	https://www.megasoftware.net/	Tamura et al.^33^
XDS (build 20220110)	https://xds.mr.mpg.de/	Kabsch^34^
Phenix 1.20.1	https://phenix-online.org/	Zwart et al.^35^
WinCoot 0.9.8.1	https://bernhardcl.github.io/coot/	Emsley et al.^36^

### Experimental model and study participant details

#### *Escherichia coli*

*Escherichia coli* strain BL21 (DE3) cells were used for heterologous expression of Castilleja *KAI2* proteins. Cells were cultured in Lysogenic Broth (LB) medium and induced with 0.1 mM IPTG at an optical density (OD600) of 0.6, followed by overnight incubation in Terrific Broth (TB) medium at 18°C.

#### *Castilleja* sp.

Seeds of all *Castilleja* species in this study were stratified on wet Whatman filter paper at 4°C in the dark for six weeks, after which they were transferred to a growth chamber maintained at 21°C under a 16-hour light/8-hour dark cycle. In GR24-treated experiments, seeds were exposed to a 1 μM concentration of GR24 5DO.

### Method details

#### *Castilleja* seed stratification and germination

*Castilleja* seeds sourced from San Diego County, California were stratified for 6 weeks on wet Whatman filter paper at 4°C in the dark. After, they were transferred for germination into a growth chamber at 21°C and a 16 h light cycle. For GR24-treated seeds, a 1 μM concentration of GR24 5DO DO (StrigoLab, Turin, Italy) was used.

#### Microscopy

*Castilleja* seedlings were directly observed on Whatman filter paper without any fixation. Images were captured using a Zeiss Axio Zoom.V16 microscope in brightfield mode, employing a 2x optovar and a 0.63x camera adapter. Images were acquired at a resolution of 2464 x 2056 pixels. Adjustments for brightness and contrast were applied using the ZEN 3.7 software (Carl Zeiss AG) to enhance clarity and detail. To assess cell viability, we used Rhodamine 123, which selectively stains mitochondria in living cells.^37^ The plants were submerged for 30 minutes in a Rhodamine 123 staining solution, prepared to a final concentration of 0.02% (w/v). Root structures and cells were then examined using a Leica Stellaris 8 confocal microscope.

#### Genomic DNA isolation

Genomic DNA was isolated using the Nanobind plant nuclei kit (PacBio). 15 μg of genomic DNA was isolated from 2 g of a single *Castilleja foliolosa* flower.

#### Genome sequencing and assembly

Sequencing libraries were prepared using the PacBio SMRTbell Express Template Prep Kit 2.0, following the manufacturer’s guidelines. Sequencing was conducted on the PacBio Sequel IIe system. The raw HiFi reads generated from the PacBio Sequel IIe platform were assembled into contigs using Hifiasm.^32^ Default parameters were used for the assembly, and quality control metrics were examined to ensure the fidelity of the assembly.

#### Gene prediction and annotation

*Castilleja foliolosa* gene prediction was performed using HELIXER^38^ and functional annotation was carried out using InterProScan^39^ and EggNOG-mapper.^33^

#### Genomic synteny analysis and phylogeny

Pairwise synteny analyses were done using SynMap^34^ with a minimum number of aligned pairs set to 20. The 'tidyverse' package^35^ in R^36^ was used to transform aligncoords files: The data file was imported, lines commencing with '#' were filtered out, and chromosome IDs were extracted from the data. Genome fasta files were transposed into segment data files using the 'Biostrings' package^40^: Lengths of the sequences were computed and a data frame containing chromosome names, start positions, and end positions were created. Link and segment data were adjusted to the actual genome sizes by applying scaling factors. The 'OmicCircos' package^41^ was then used to generate a Circos plot, and links indicating associations between segments were added.

A phylogenetic tree of KAI2 protein sequences was built using the Maximum Likelihood method and JTT matrix-based model. The tree with the highest log likelihood is shown. The final tree was displayed using MEGA11.^42^

#### Molecular cloning

For heterologous protein production, all genes were synthesized codon-optimized for *Escherichia coli* and cloned into a pGEX 4T1 expression vector to produce GST fusion proteins. Genes were designed to encode an N-terminal site for HRV3 protease, leaving two amino acids (Gly-Pro) as N-terminal cloning artifact.

#### Protein expression and purification

*Escherichia coli* strain BL21 (DE3) cells were transformed and subsequently cultivated overnight in Lysogenic Broth (LB) medium. A fresh culture was initiated in Terrific Broth (TB) medium the next day using a 1:100 dilution. Cultivation was maintained at 23°C until an optical density (OD_600_) of 0.6 was reached, after which induction was performed with 0.1 mM isopropyl β-D-1-thiogalactopyranoside (IPTG) at 18°C and left overnight. The cells were harvested, and lysis was achieved through sonication. Centrifugation at 75,000 g for 45 minutes was then used to separate cell debris, after which the supernatant was transferred onto a glutathione affinity column. This column was flushed with a buffer of 50 mM TRIS-HCl, 150 mM NaCl, 5% glycerol, and 1 mM TCEP, with a final pH of 7.7, until no protein flow-through was detected by UV absorption. HRV3 protease was subsequently introduced to the column and left overnight. The cleaved target protein was eluted using the same buffer and further purified to homogeneity through size exclusion chromatography on a GE Healthcare HiLoad 16/60 Superdex 75 column, prepared with 20 mM TRIS-HCl, 30 mM NaCl, and 1 mM TCEP-HCl buffer, with a final pH of 7.7. The proteins were concentrated to a minimum of 10 mg/ml and flash-frozen in liquid nitrogen.

#### Differential scanning fluorimetry

Differential scanning fluorimetry experiments were performed in a CFX Opus 384 system (Biorad). Sypro Orange (Life Technologies) was used as reporter. 10 μg of protein was heat-denatured using a linear 25°C to 95°C gradient at a rate of 1°C per minute. The denaturation curve and its derivative were obtained using the CFX manager software. Reaction mixtures were prepared in 20 μl volumes in triplicates in 384 well white microplates. Reactions were carried out in 20 mM TRIS-HCl, 30 mM NaCl, 1 mM TCEP-HCl, final pH 7.7. A final 3x concentration of Sypro Orange was used.

#### Protein crystallization and structure solution

Crystals of *Castilleja foliolosa* KAI2d15 were obtained from a 200 nl sitting drop, prepared with a protein-to-reservoir ratio of 1:1 and a chemical composition of 0.9 M diammonium phosphate, 0.1 M sodium acetate at pH 4.5, and 0.01 M barium chloride. To these crystals, 1.8 M sodium malonate was applied as a cryo-protectant. X-ray data were collected at beamline 8.2.2 of the Advanced Light Source at Lawrence Berkeley National Laboratory and subsequently processed with XDS.^43^ The structure of KAI2d15 was solved with Phaser through molecular replacement, utilizing chain A of PDB structure 4IH1 (*Arabidopsis thaliana* KAI2) as the model. For the calculation of R-free, five percent of the data were flagged. Initial models were constructed using AutoBuild and refinement of the models was performed using phenix.refine, all of which are part of the Phenix suite.^44^ Manual correction and finalization of these models were accomplished with Coot.^45^

### Quantification and statistical analysis

Data for seed germination, DSF destabilization, and Michaelis-Menten kinetics are presented as mean values with standard deviation (SD) across three or more independent experiments and calculated with R for seed germination and with Origin (OriginLab) for DSF and Michaelis-Menten kinetics. Detailed sample sizes (n) and specific conditions are noted in each figure legend, along with any variation among replicates, with error bars representing the standard deviation where applicable.
