# Supplementary material for: Strigolactones initiate the formation of haustorium-like structures in Castilleja
Source: iScience. 2024 Nov 28;27(12):111491. doi: 10.1016/j.isci.2024.111491 (PMC11697714; doi:10.1016/j.isci.2024.111491)
Supplement: Document S1. Figures S1–S4 and Tables S1–S3 [file mmc1.pdf]

iScience, Volume *27*

Supplemental information

Strigolactones initiate the formation of haustorium-like structures in *Castilleja*

Marco Bürger, Danica Peterson, and Joanne Chory

Table S1. Genome assembly statistics of *Castilleja foliolosa*. Related to Figure 2. Statistics were generated using the ‘assembly-stats’ software.

Sum	751,543,112 bp	
n contigs	551	
Average contig length	1,363,962 bp	
Largest contig	32,005,117 bp	
N50	11,150,047 bp	n = 20
N60	9,119,997 bp	n = 28
N70	6,703,553 bp	n = 37
N80	4,840,700 bp	n = 51
N90	2,884,952 bp	n = 70
N100	5,772 bp	n = 551
‘N’ count	0	
Gaps (intra-contig)	0	

Table S2. BUSCO analysis of the *Castilleja foliolosa* genome annotation. Related to Figure 2. Busco version 5.3.2 was used against the eudicots_odb10 lineage dataset with the ‘hmmsearch’ 3.1 dependency.

Total BUSCO groups searched	2326
Complete BUSCOs	2033 (87.5%)
Complete and single-copy BUSCOs	1655 (71.2%)
Complete and duplicated BUSCOs	378 (16.3%)
Fragmented BUSCOs	109 (4.7%)
Missing BUSCOs	184 (7.8%)

Table S3. ***C. foliolosa*** **KAI2d15 data collection and refinement statistics.** Related to Figure 3. Statistics for the highest-resolution shell are shown in parentheses.

Wavelength (Å)	1.0000
Resolution range (Å)	69.54 – 1.80 (1.86 – 1.80)
Space group	$P\ 2_1\ 2\ 2_1$
Unit cell (°)	59.58, 69.54, 156.56, 90.00, 90.00, 90.00
Total reflections	729883 (38881)
Unique reflections	60424 (6013)
Multiplicity	12.1 (7.1)
Completeness (%)	99.95 (99.97)
Mean I/sigma(I)	16.23 (0.76)
Wilson B-factor (Å²)	26.20
R-merge	0.07192 (0.4146)
R-meas	0.07494 (0.4462)
CC1/2	0.999 (0.931)
CC*	1 (0.982)
R-work	0.2124 (0.3880)
R-free	0.2618 (0.3970)
Number of non-hydrogen atoms	4612
macromolecules	4204
ligands	24
solvent	384
Protein residues	536
RMS(bonds) (Å)	0.006
RMS(angles) (°)	1.00
Ramachandran favored (%)	98.13
Ramachandran allowed (%)	1.50
Ramachandran outliers (%)	0.37
Clashscore	9.03
Average B-factor (Å²)	31.09
macromolecules	30.69
ligands	41.66
solvent	34.80

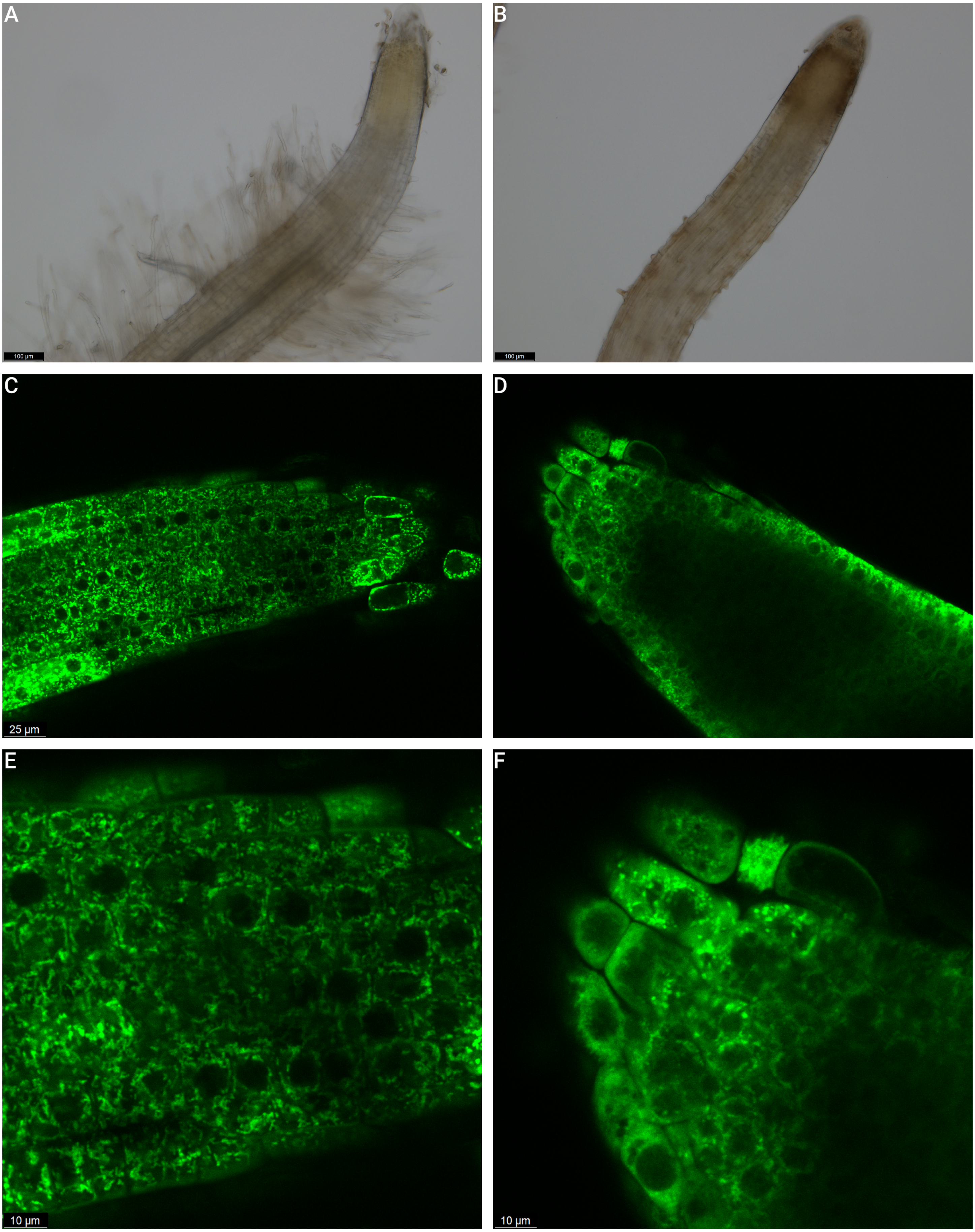
A
100 µm
B
100 µm
C
25 µm
D
E
10 µm
F
10 µm

Figure S1. *Castilleja foliolosa* root structures. Related to Figure 1. **(A)** Root of a plant germinated in H_2O. **(B)** Root of a plant germinated in the presence of GR24 5DO. **(C,E)** Rhodamine 123 stained root of a plant germinated in H_2O. **(D,F)** Rhodamine 123 stained root of a plant germinated in the presence of GR24 5DO.

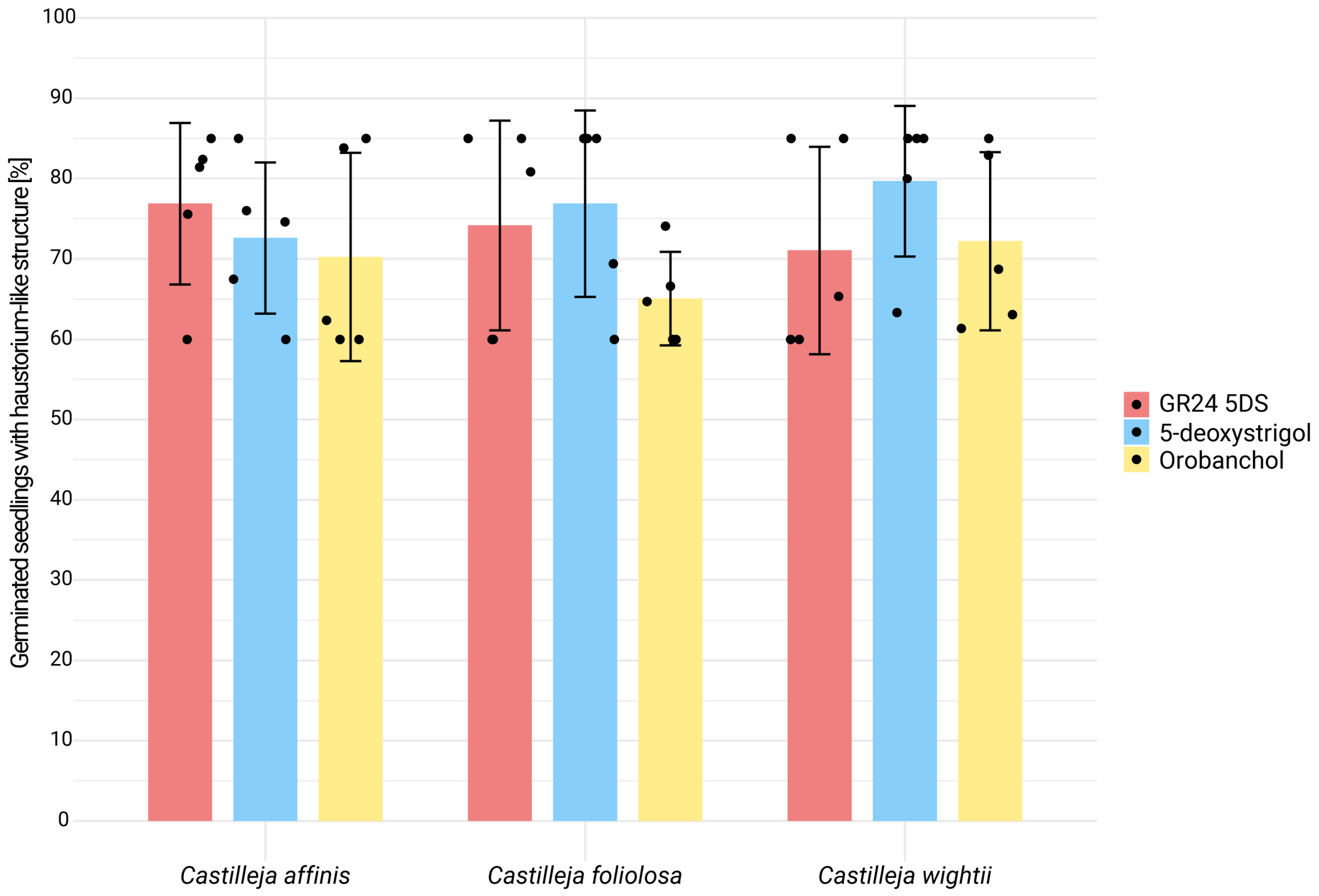

Figure S2. Percentage of seedlings with haustorium-like root structure after application of different strigolactones. Related to Figure 1.

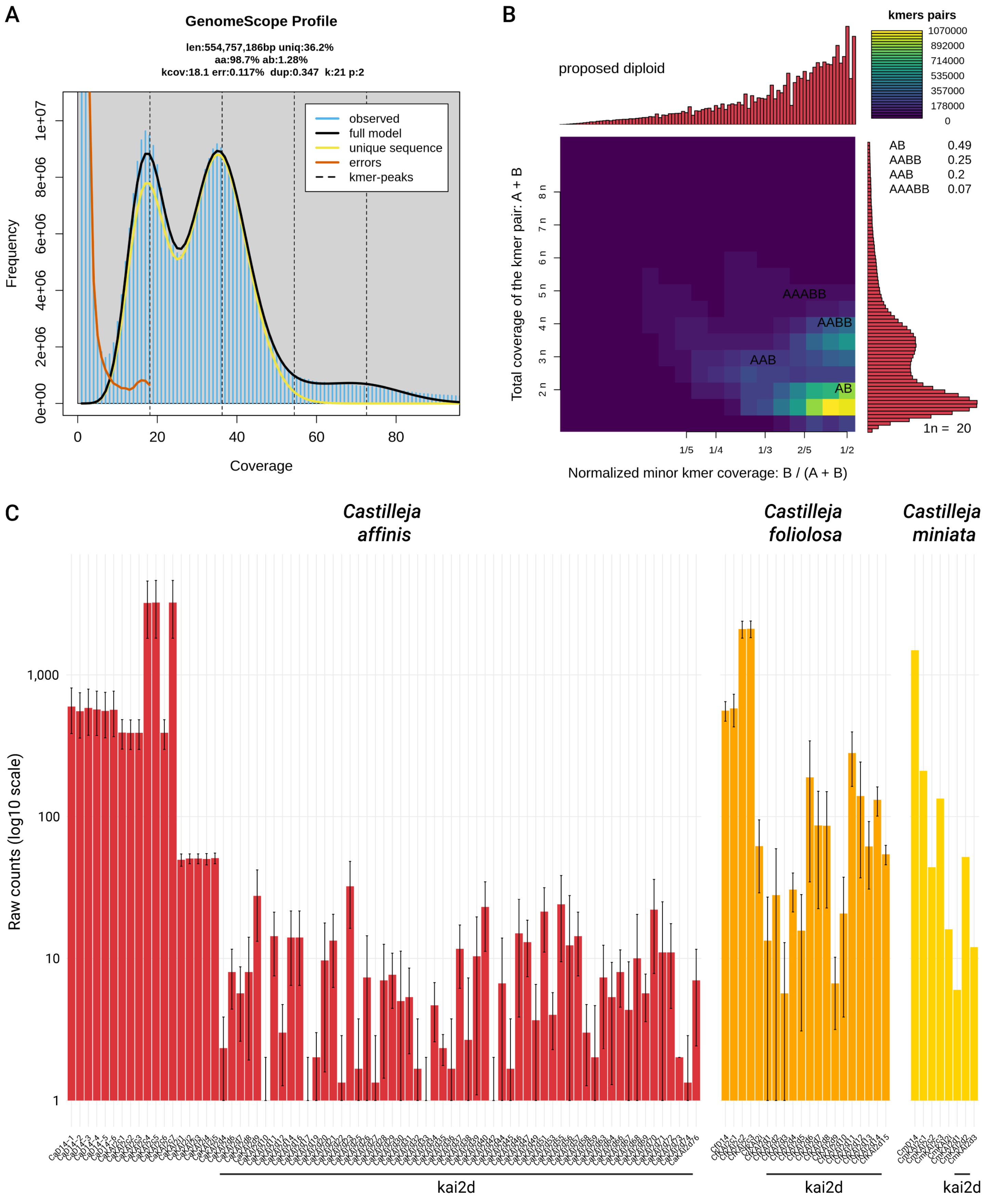

Figure S3. K-mer based analysis of the *Castilleja foliolosa* genome assembly. Related to Figure 2. **(A)** GenomeScope profile indicating an 18x sequencing coverage and an estimated haploid length

of 555 Mbp. **(B)** Smudgeplot analysis indicating a diploid genome. **(C)** Presence of different kai2d ortholog transcripts in different *Castilleja* species. Bulk RNA from *Castilleja affinis* and *Castilleja foliolosa* seedlings were sequenced using PacBio Iso-seq, and a public RNAseq dataset from *Castilleja miniata* (Accession: SRX8528012) was included.

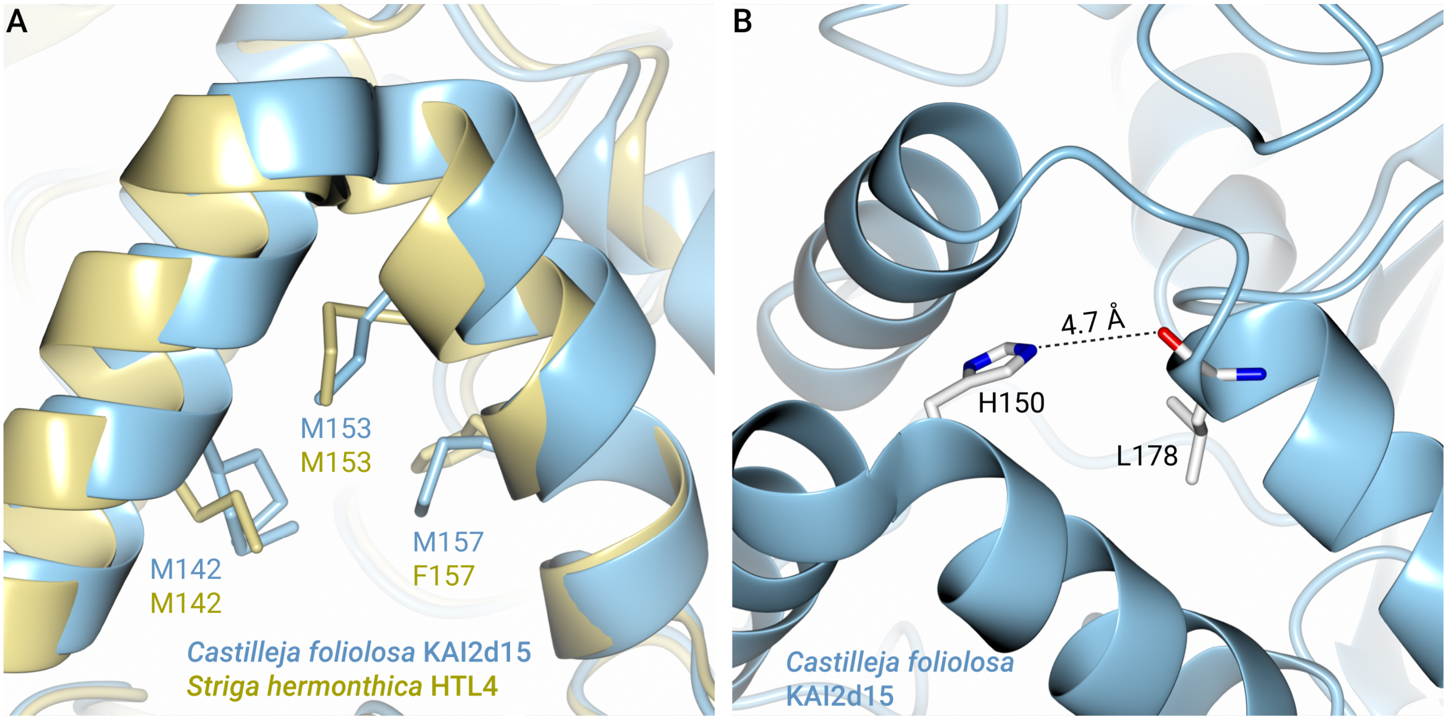

Figure S4. Comparison of the *Castilleja foliolosa* KAI2d15 and *Striga hermonthica* HTL4 crystal structures. Related to Figure 3. **(A)** Lid-domains showing the residues surrounding the entrance to the substrate binding pocket. **(B)** The distance between H150 and L178 in *Castilleja foliolosa* KAI2d15.
